# Carbon Nanofibers Decorated by MoS_2_ Nanosheets with Tunable Quantity as Self-Supporting Anode for High-Performance Lithium Ion Batteries

**DOI:** 10.3390/nano13192689

**Published:** 2023-09-30

**Authors:** Liyan Dang, Yapeng Yuan, Zongyu Wang, Haowei Li, Rui Yang, Aiping Fu, Xuehua Liu, Hongliang Li

**Affiliations:** 1 State Key Laboratory of Bio-Fibers and Eco-Textiles, Institute of Materials for Energy and Environment, College of Materials Science and Engineering, Qingdao University, Qingdao 266071, China; 2 College of Chemistry and Chemical Engineering, Qingdao University, Qingdao 266071, China

**Keywords:** carbon nanofibers, MoS_2_ nanosheets, core//sheath structure, electrospinning, self-supporting anode

## Abstract

Two-dimensional molybdenum disulfide (MoS_2_) is considered as a highly promising anode material for lithium-ion batteries (LIBs) due to its unique layer structure, large plane spacing, and high theoretical specific capacity; however, the overlap of MoS_2_ nanosheets and inherently low electrical conductivity lead to rapid capacity decay, resulting in poor cycling stability and low multiplicative performance. This severely limits its practical application in LIBs. To overcome the above problems, composite fibers with a core//sheath structure have been designed and fabricated. The sheath moiety of MoS_2_ nanosheets is uniformly anchored by the hydrothermal treatment of the axial of carbon nanofibers derived from an electrospinning method (CNFs//MoS_2_). The quantity of the MoS_2_ nanosheets on the CNFs substrates can be tuned by controlling the amount of utilized thiourea precursor. The influence of the MoS_2_ nanosheets on the electrochemical properties of the composite fibers has been investigated. The synergistic effect between MoS_2_ and carbon nanofibers can enhance their electrical conductivity and ionic reversibility as an anode for LIBs. The composite fibers deliver a high reversible capacity of 866.5 mA h g^−1^ after 200 cycles at a current density of 0.5 A g^−1^ and maintain a capacity of 703.3 mA h g^−1^ after a long cycle of 500 charge–discharge processes at 1 A g^−1^.

## 1. Introduction

With the increase in energy demands and the rapid consumption of non-renewable resources, especially fossil energy, energy shortage techniques have become a crucial point for the application of sustainable resources [1,2]; therefore, the search for new green energy that is friendly to the environment and the development of more advanced energy storage technologies have attracted intense attentions around the world [3,4,5,6]. The development of more stable, efficient and low-cost energy storage technologies is the key issue for solving the energy problem [7,8]. Lithium-ion batteries (LIBs) are the most promising energy storage agency for portable electronic devices and electric vehicles owing to their wide electrochemical window, long cycle life and high energy density [9,10,11,12,13]. Graphite, as the most widely used anode material, possesses the advantages of abundance, low cost and a long lifetime, but its application in LIBs is hindered by its low theoretical capacity (372 mAh g^−1^) and poor rate performance [14,15]. Therefore, the development of an alternative as an anode with a high capacity, high energy density, long cycle stability and excellent rate capability is the key issue for high-performance LIBs. Various transition metal sulfides (TMSs) have been considered as promising anode materials because of their unique layered structure, large specific surface area and high theoretical specific capacity [16,17]. In particular, layered MoS_2_ with an intercalated structure has received wide attention due to its hierarchical nanostructure and physicochemical properties [18,19]. MoS_2_ is assembled from three layers of atoms (S-Mo-S) through weak van der Waals interactions, with Mo atoms sandwiched between two layers of S atoms, and this intercalated structure could facilitate the rapid insertion of Li^+^ ions [20]; however, similar to many transition metal disulfides, the commonly used MoS_2_ anodes for LIBs also face two crucial bottlenecks, i.e., (1) the huge mechanical strain and volume change during repeated discharge/charge could lead to electrode crushing, resulting in rapid capacity decay [21,22]; and (2) the low conductivity of MoS_2_ nanosheets causes rapid capacity decay and poor rate capability [23,24]. The endeavors to overcome such problems mainly are (1) to increase the atomic layer spacing to accelerate the lithiation process and reduce the damage of the MoS_2_ nanostructure during charge/discharge cycles, and (2) to combine MoS_2_ with carbon-based materials to improve its electronic/ionic conductivity [22,25,26,27,28]. For example, MoS_2_ nanoflowers prepared by Xu et al. presented a reversible capacity of 93.6 mAh g^−1^ at a current density of 50 mA g^−1^ during lithium-ion battery cycling [29]. Meanwhile, MoS_2_ grown on reduced graphene oxide with large layer spacing achieved a reversible capacity of 450 mAh g^−1^ at 100 mA g^−1^. As a kind of one-dimensional carbon material, carbon nanofibers derived from electrospinning not only possess excellent electrical conductivity, but also own good flexibility as a substrate. More importantly, carbon nanofibers can be used as a conductive agency to replace the commonly used binders and carbon black additives, which can effectively improve ion/electron transport and thus exhibit excellent electrochemical properties [3,24,30].

In this paper, the self-supporting substrate of carbon nanofibers are firstly prepared by the electrostatic spinning method, and then MoS_2_ sheets are uniformly anchored on the surface of the carbon nanofiber by a simple solvothermal method. The resulted CNFs//MoS_2_ composite nanofibers with a core//sheath structure can be used as a self-standing electrode for LIBs, which simplify the electrode preparation process. The composite structure can effectively alleviate the pulverization of the anchored MoS_2_ sheets upon charge and discharge. Meanwhile, the rigid skeleton of the CNFs network could promote the rapid ion/electron transfer in the CNFs//MoS_2_ composite anode and improve the capacity and electrical conductivity. Benefited by the synergistic effect between the two moieties of CNFs and MoS_2_, the optimized composite fiber anode delivers a high reversible capacity of 866.5 mA h g^−1^ after 200 cycles at a current density of 0.5 A g^−1^ and the good cycling stability of 500 cycles at 1 A g ^−1^, maintaining a capacity of 703.3 mA h g^−1^.

## 2. Experimental Section

### 2.1. Materials

Sodium molybdate dihydrate (Na_2_MoO_4_•2H_2_O), Thiourea (CH_4_N_2_S), Glucose (C_6_H_12_O_6_), Polyacrylonitrile (PAN), N, N-Dimethylformamide (DMF), Ethanol, and deionized water are used as received without further purification.

### 2.2. Preparation of CNFs

First, 0.3 g of PAN and 3ml of DMF were heated in a 40 °C water bath to obtain the precursor solution, and then the DMF solution of PAN was loaded into a syringe for electrospinning. The spinning voltage was set as 14 KV, and the receiving distance of 15 cm and the flow rate of 0.14 μL min^−1^ were applied. The PAN nanofibers were coated on a silicone oil paper after the spinning, and then the PAN film was pre-oxidized at 240 °C for 2 h in a muffle furnace at a heating rate of 2 °C per min. Finally, the film was carbonized at 550 °C for 3 h in an inert atmosphere of Ar with a heating rate of 5 °C a minute.

### 2.3. Synthesis of the Core//Sheath Structured CNFs//MoS_2_ Composite Fibers

A total of 0.1 g of Na_2_MoO_4_•2H_2_O was added to 25 mL of 0.05 mmol/L of glucose solution and stirred until completely dissolved. A certain amount of thiourea was added to the solution and stirred until completely dissolved. The solution was then transferred to a 50 mL Teflon-lined autoclave in which a piece of aforementioned CNFs film with size of 2 cm × 2 cm was input in advance. The autoclave was treated at 200 °C for 24 h. Finally, it was naturally cooled to room temperature and solid was obtained by centrifugation. The solids were washed three times with deionized water and ethanol and then they were dried at 70 °C for 6 h. Thereafter, they were heated to 600 °C for 3 h at 5 °C a minute in H_2_/Ar atmosphere to crystalize the MoS_2_ moiety. Samples named as CNFs//MoS_2_-0.05, CNFs//MoS_2_-0.1, CNFs//MoS_2_-0.15, CNFs//MoS_2_-0.2, CNFs//MoS_2_-0.3 and CNFs//MoS_2_-0.4, respectively, were derived, in which the numbers 0.05, 0.1, 0.15, 0.2, 0.3 and 0.4 correspond to the quantity of utilized thiourea in grams for the preparation.

### 2.4. Materials Characterizations

The structure of the product was characterized by Rigaku Ultima IV X-ray diffractometer (XRD, Cu Ka radiation k = 0.15318 nm). The morphology and structures were characterized by JEOL JSM-7800 F field emission scanning electron microscope (FESEM). Energy dispersive spectroscopy (EDS) was performed with the Oxford attachment. JEOL JEM-2100 transmission electron microscope (TEM) was used for structure observation. Raman spectra were obtained on a confocal laser micro-Raman spectrometer (renishaw). The X-ray photoelectron spectroscopy (XPS) was obtained by the PHI 5000 Versaprobe III XPS. Nitrogen sorption isotherms were recorded by an Autosorb IQ MP/XR surface area and pore analyzer (Quantachrome). The surface area was calculated by the Brunauer–Emmetteller (BET) model and the pore size distribution was estimated using the Barrett–Joyner–Halenda (BJH) method with the adsorption branch. The thermal stability of the materials was effectively characterized by a METTLER TOLEDO TGA-2 thermogravimetric analyzer (TGA) with a temperature of 25–800 °C under atmosphere of air with a heating rate of 10 °C min^−1^. 

### 2.5. Electrochemical Characterizations

The film of composite fiber was cut into discs with a diameter of about 10 mm by a slicing machine, and the accurate weight of each electrode was measured. LiPF_6_ with a concentration of 1 mol/L in a mixture of ethylene carbonate (EC)/dimethyl carbonate (DEC)/diethyl carbonate (DMC) with a volume ratio of 1:1:1 were used as the electrolyte. Celgard 2400 film was used as the separator and the lithium foil was used as the counter electrode. The coin cell of 2016 type was fixed in a glove box filled with high-purity argon. The assembled cells were statically activated for 10 h. Then the electrochemical performance was measured by the 2001 A battery tester (Land, Wuhan, China) in the cut-off voltage range of 3~0.01 V. Cyclic voltammetry (CV) was performed by the CHI 760D electrochemical workstation in a voltage range of 0.01~3 V with a scanning rate of 0.1 mV s^−1^. In addition, electrochemical impedance spectroscopy (EIS) was performed with the same electrochemical workstation. The frequency range is set between 100 kHz and 0.01 Hz and the amplitude is set as 5 mV. 

## 3. Results and Discussion

**Figure 1:**
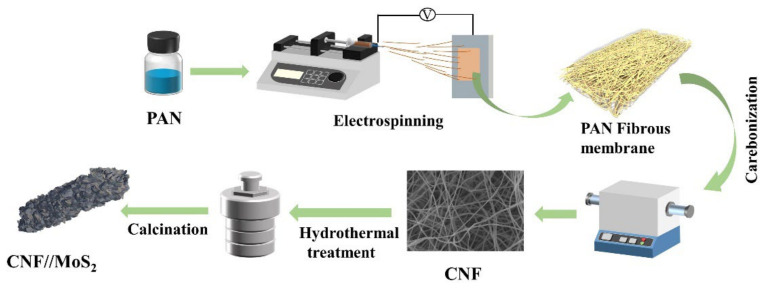
Schematic illustration of the fabrication process for the CNFs//MoS_2_ composite fibers.

Figure 1 shows the synthesis process of the CNFs//MoS_2_ composite films. Firstly, the precursor of the carbon nanofiber, i.e., PAN nanofibers, is prepared by electrospinning and is then converted to CNFs after a calcination step. MoS_2_ is uniformly anchored on the surface of CNFs by a simple solvothermal method. Thiourea is used as a source to produce S^2−^. Finally, CNFs//MoS_2_ composite fibers with flexible and self-supporting properties are synthesized after a crystallization process.

**Figure 2:**
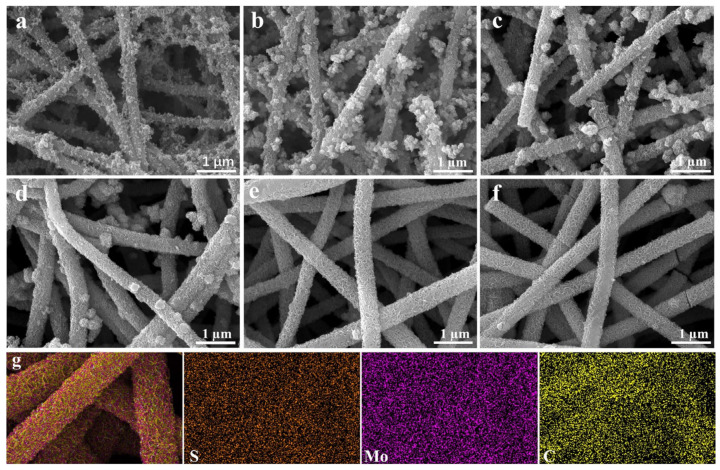
SEM images of (**a**) CNF//MoS_2_-0.05, (**b**) CNF//MoS_2_-0.1, (**c**) CNF//MoS_2_-0.15, (**d**) CNF//MoS_2_-0.2, (**e**) CNF//MoS_2_-0.3, (**f**) CNF//MoS_2_-0.4 and (**g**) EDS elemental mappings for CNF//MoS_2_-0.3.

It can be seen from Figure 2 that the morphologies of the resulted CNFs//MoS_2_ composite fibers change from rough surfaces (Figure 2a,b) to more homogenous ones (Figure 2a,b) with the increase in the thiourea content for the preparation. For sample CNF//MoS_2_-0.05, the MoS_2_ sheets are anchored randomly on the CNFs substrates and the latter are not covered completely with MoS_2_ species. The dispersion of MoS_2_ nanosheets in CNF//MoS_2_-0.1 becomes denser than that in CNF//MoS_2_-0.05, however, extruded MoS_2_ particles with much larger sizes still can be observed on the surface of CNFs for the former (Figure 2b). With the increase in the thiourea quantity, the numbers of extruded MoS_2_ particles on the surface of CNFs decrease (Figure 2c,d). When the addition amount of thiourea reaches 0.3 g (the mass ratio of Na_2_MoO_4_: thiourea = 1:3), the resulted sample of CNF//MoS_2_-0.3 shows the homogeneous deposition of MoS_2_ sheets on the surface of the CNFs substrates (Figure 2e). In comparison with CNF//MoS_2_-0.3, the CNF//MoS_2_-0.4 sample displays no obvious difference in morphology, indicating that the influence of the thiourea content to the morphology becomes rigid after that (Figure 2f). Another variation with the increase in thiourea is the diameters of the composite fibers, which increases from roughly 200 nm for CNF//MoS_2_-0.05 to about 300 nm for CNF//MoS_2_-0.3. The increase in diameter for the CNF//MoS_2_ composites can be ascribed to the continuous growth of MoS_2_ nanosheets on the surface of the CNFs substrates. It has been reported that the hydrolysis of thiourea can be tuned by changing the concentrations of the added NaOH solution, as indicated with Equation (1). The high concentration of the NaOH solution will accelerate the hydrolysis of thiourea and prompt the supply rate of S^2−^ ion. The sodium molybdate can be reduced by S^2−^ and the resulted Mo^4+^ cations then combine with S^2−^, following Equation (2). Therefore, MoS_2_ microspheres with different morphologies from loose nanoflowers to compact spherical structures can be obtained when NaOH solutions of varied concentrations have been applied [31].

(NH_2_)_2_SC + OH^−^ → S^2−^ + CH_2_N_2_ + H_2_O (1)

MoO_4_^2−^ + S^2−^ → MoS_2_ + SO_4_^2−^ (2)

In this work, we try to control the quantity of the resulted MoS_2_ nanosheets on the CNFs substrate by tuning the content of thiourea instead of the NaOH solution (actually, no NaOH has been applied in our perpetration). With the increase in the quantity of thiourea, more S^2−^ can be supplied over the same period. Therefore, it can be inferred that when 0.3 g of thiourea is applied in the preparation, the supply of S^2−^ is no longer the limitation of the total reaction rate. At a low thiourea concentration, the supply of S^2−^ might become the rate limiting step, which retards the reaction of Equation (2). Therefore, a deposit of MoS_2_ with different morphologies and contents can be derived in this work. The variation of mass densities from pristine CNFs to CNF//MoS_2_-X composite fibers confirms the SEM observation, e.g., mass densities of 0.100 g/cm^−3^ for CNFs and 0.103, 0.105, 0.110, 0.113, 0.155 and 0.159 g/cm^−3^ for CNF//MoS_2_-0.05, -0.01, -0.15, -0.2, -0.3 and -0.4, respectively, have been calculated. When 0.4 g of thiourea is utilized, the morphology of the resulted CNF//MoS_2_-0.4 is almost the same as that of CNF//MoS_2_-0.3, which means the influence of the thiourea concentration turns weak after the point of 0.3. The EDS mapping images (Figure 2g) show the uniform distribution of the Mo, S and C elements in the composite fibers.

**Figure 3:**
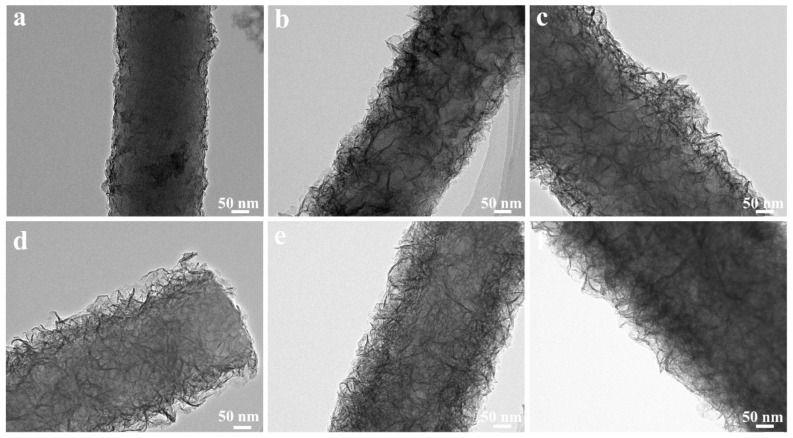
TEM images of (**a**) CNF//MoS_2_-0.05, (**b**) CNF//MoS_2_-0.1, (**c**) CNF//MoS_2_-0.15, (**d**) CNF//MoS_2_-0.2, (**e**) CNF//MoS_2_-0.3, (**f**) CNF//MoS_2_-0.4.

**Figure 4:**
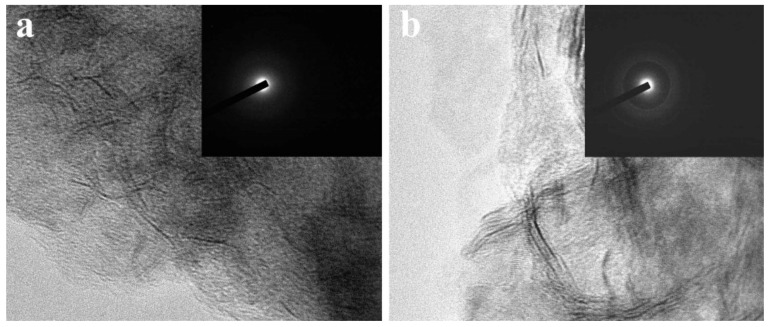
The HRTEM images of (**a**) CNF//MoS_2_-0.05 and (**b**) CNF//MoS_2_-0.3, the insets show their SAED patterns.

TEM measurements have been carried out to investigate the structure evolution from CNF//MoS_2_-0.05 to CNF//MoS_2_-0.4. As can be seen from Figure 3, the density of the MoS_2_ nanosheets on the surface of CNFs substrates becomes more obvious with the increase in the applied thiourea content. The CNF//MoS_2_-0.05 shows a very thin layer of MoS_2_ nanosheets coated on the surface of CNFs. The diameter of the composite fiber is about 250 nm (Figure 3a). Obviously, the MoS_2_ nanosheets on the CNF//MoS_2_-0.1 sample become more observable and the diameter of the composite fiber increases slightly (Figure 3b). With the increase in the thiourea content from 0.15 to 0.4 g for the preparation, one can see that the MoS_2_ nanosheets become more and more compact in the corresponding composite fibers (see Figure 3c–e). For CNF//MoS_2_-0.3, a dense layer of MoS_2_ nanosheets with a thickness of about 50 nm is uniformly anchored on the CNFs substrate and a boundary between the CNF core and MoS_2_ coating layer can be roughly observed (Figure 3e). A similar result has been obtained for the CNF//MoS_2_-0.4 sample (Figure 3f), which is also coated by a compact layer of MoS_2_ nanosheets. The TEM images are consistent with those observed by SEM (Figure 2). High-resolution TEM observations have also been performed by focusing the edges of the composite fibers. As shown in Figure 4a, only a few MoS_2_ layers can be observed on the surface of the CNF//MoS_2_-0.05 sample. In contrast, the CNF//MoS_2_-0.01 sample and the other ones prepared with a high thiourea content present clear S-Mo-S layers with large interlayer spacing (Figure S1b–d and Figure 4b). The HRTEM images herein show clearly the curved stripe of MoS_2_, which is consistent with the TEM images of Figure 3 in which twisted MoS_2_ nanosheets anchored on the surface of CNFs are observed. As can be seen from their selected area electron diffraction patterns (see the inserts in Figure S1 and Figure 4), very weak reflection rings can be observed for the samples, except for CNF//MoS_2_-0.05. Such a kind of SAED character coincides with the irregular stacking of the MoS_2_ layers.

**Figure 5:**
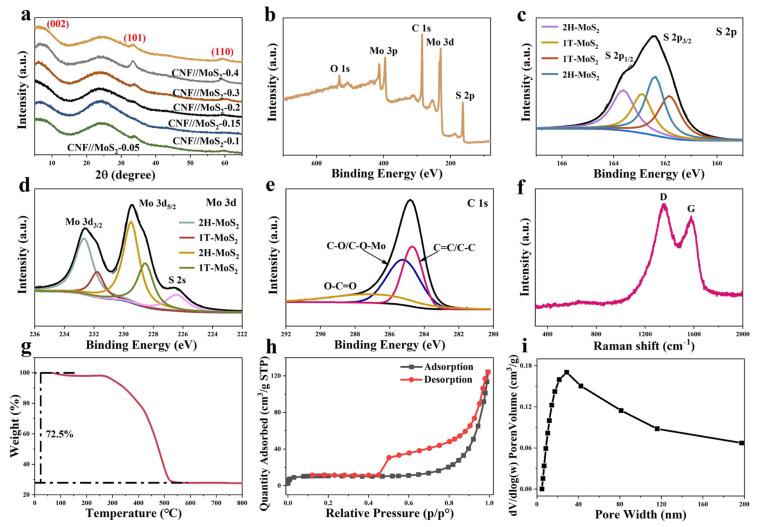
(**a**) XRD patterns of these sample, and (**b**) XPS full survey and high-resolution XPS spectra of (**c**) S2p, (**d**) Mo3d, (**e**) C1s for CNF//MoS_2_0.3, (**f**) Raman spectra, (**g**) TGA curve, (**h**) the N_2_ sorption isotherms and (**i**) pore-size distribution curve of CNF//MoS_2_0.3.

As shown in Figure 5a, the MoS_2_ prepared herein is different from the common one of JCPDS 77-1716; it is the 2H-type MoS_2_. No (002) crystal plane reflection at 2θ = 14° which can be observed in the 2H configuration of the sample, but a strong (002) crystal plane reflection peak near 7° appears due to the expansion of the lattice spacing [32,33]. In comparison with the normal 2H-MoS_2_ structure, the spacing can be expanded from 0.62 nm to 1.30 nm for the MoS_2_ obtained in this work, which may be due to the intercalation of molecules or ions in between the planes of the two-dimensional MoS_2_ [33,34,35], and similar phenomena have also been observed for MoS_2_ prepared by solvothermal processes [33,36]. The full XPS survey and high-resolution ones of CNF//MoS_2_-0.3 are shown in Figure 5. From panel 5b it can be seen that the peak of the O element is relatively lower than that of other elements, indicating that the most of the oxygen-containing functional groups decomposed during the carbonization process. High-resolution XPS for elements of S, Mo and C, respectively, are detected and analyzed to learn more details on them. The S2p peak at 162.5 in Figure 5c can be deconvoluted into two peaks at 163.7 and 162.4 eV, corresponding to S2p_1/2_ and S2p_3/2_, respectively, of divalent sulfur and confirming the status of the sulfur in MoS_2_. From Figure 5d it can be seen that two Mo^4+^-related peaks at a binding energy of 232.6 for Mo3d_3/2_ and 229.5 eV for Mo3d_5/2_ can be detected, proving the IV state of Mo in the composite fibers [37]. Furthermore, both the S2p_3/2_ and the Mo3d_5/2_ can be deconvoluted deeply into the 1T and the 2H phases of MoS_2_, as depicted in panels a and d of Figure 5. The C ls peak, which appears around 284.8 eV, can be deconvoluted into three peaks (see Figure 5e), i.e., the first one at 284.6 eV corresponding to the carbon–carbon single bond of carbon materials, the second peak at 285.3 eV, which can be ascribed to the carbon element in the C-OH/C-O-Mo status, and the third one related to the C=O connection at 287 eV [38,39].

To gain more structure properties of the carbon species, Raman spectra are recorded for the sample of CNF//MoS_2_-0.3. As shown in the Figure 5f, two obvious peaks at ~1334 and ~1578cm^−1^ can be observed. The former is called the D-band-related peak, which is attributed to the defects or structure disordering of graphite. The latter is called the G-band peak, which corresponds to the pristine sp^2^ bond of carbon in graphite [40]. The ratio of intensity for the D-band to G-band, i.e., ID/IG, is usually used to explain the degree of the graphitization of carbon materials. The value of ID/IG for the CNF//MoS_2_-0.3 sample is about 1.09, indicating that the degree of graphitization is reasonable. However, the MoS_2_-related characteristic peaks in between 380 and 410 cm^−1^ have not been recorded in the spectrum, which might be due to the dislocation or disordering of the MoS_2_ layers. To know the thermal stability and to determine the content of carbon in the composite fibers, TGA tests have been carried out for them in an air atmosphere at a heating rate of 10 °C min^−1^. As can be seen in Figure 5g and Figure S2, there is a slight mass loss from room temperature to 150 °C, which might be the evaporation of water adsorbed in the sample. From 270 to 800 °C, a significant mass loss is detected due to the combustion of carbon in the air. By considering the transformation of MoS_2_ to MoO_3_ [41], the calibrated MoS_2_ contents in these composite fibers from CNF//MoS_2_-0.05 to CNF//MoS_2_-0.4 are approximately 17.2%, 24.6%, 28.1%, 29.0%, 30.6% and 32.2%, respectively. The MoS_2_ content in CNF//MoS_2_-0.3 is higher than those of the several reported pieces of literature [30,42]. To gain the surface area and pore size distribution, N_2_ sorption measurements have been performed. The isothermal curves and pore size distribution of CNF show only a small amount of large pores due to the folding of fibers (Figure S3). In contrast, as has been shown in Figure 5h, the typical isothermal curves of CNF//MoS_2_-0.3 show type III sorption isotherms with an obvious hysteresis lops in between the relative pressure of 0.42 and 0.92, indicating the existence of meso-sized pores. The Figure 5i confirms the presence of mesopores in the composites and a wide pore size distribution from several nanometers to more than 40 nm can be observed. A specific surface area of 32.7 m^2^ g^−1^ has been calculated by using the BET model, which is higher than the area of 20.0 m^2^ g^−1^ for pristine CNFs. The high specific surface areas and the presence of mesosized pores can be attributed to the decoration of MoS_2_ nanosheets on the surface of CNFs. The porous structure and high specific surface area would be expected to accelerate ion diffusion and improve energy storage performance.

**Figure 6:**
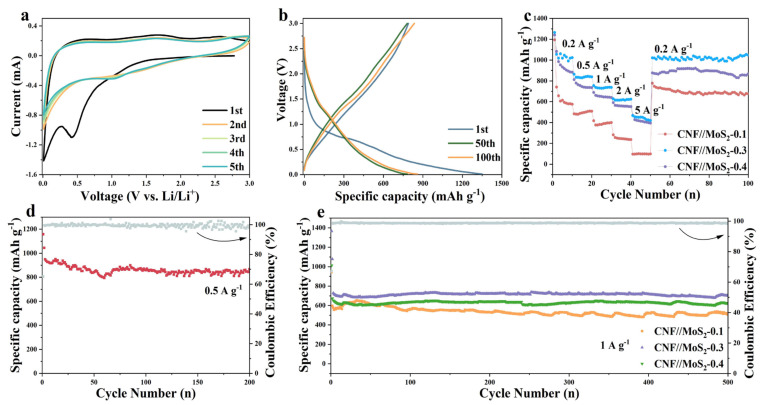
(**a**) CV profiles with a scanning rate of 0.2 mV s^−1^ and (**b**) GCD profiles at 0.5 A g^−1^ for CNF//MoS_2_-0.3. (**c**) Rate performance at different current densities for three samples. (**d**) Cycling performance of CNF//MoS_2_-0.3 at 0.5 A g^−1^ and (**e**) that at 1 A g^−1^ for three different samples.

**Figure 7:**
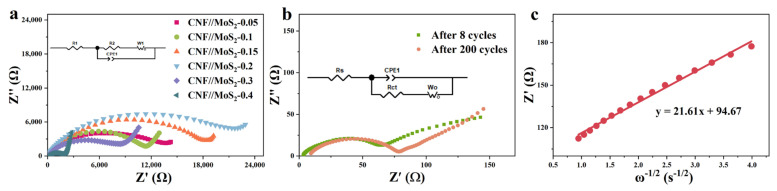
(**a**) Nyquist plots of these prepared samples, (**b**) Nyquist plots of CNF//MoS_2_-0.3 after 8 and after 200 cycles charge–discharge cyclesand (**c**) the relationship between Z’ and ω^−1/2^ in the low-frequency region after 200 cycles.

The electrochemical properties of the composite fibers and pristine CNFs have been investigated. As shown in Figures S4 and S5 and Figure 6a, in the first discharge cycle, a significant reduction peak appeared around 0.55 V, which is due to the formation of SEI film, along with the further reaction of Li_x_MoS_2_ by the formula of Li_x_MoS_2_ + (4 − x) Li^+^(4 − x)e^−^ → Mo + 2Li_2_S) for composite fibers [43]. The disappearance of the peak in the subsequent scan can be attributed to an irreversible reaction. The peak corresponding to the insertion of Li^+^ into the layer-structured lattice of Li_x_MoS_2_ has not been observed, which might be due to the enlarged inter-plane spacing of the MoS_2_ nanosheets. The peak which appeared at about 1.7 V during the subsequent discharge cycles is considered to be the shape of Li_2_S. The oxidation peak at about 1.48 V is related to the conversion of Mo to Mo^4+^, while the other oxidation peak at 2.5 V may be related to the oxidation of Li_2_S (Li_2_S → 2Li^+^ + S + 2e^−^). Obviously, these two peak have not been detected for pristine CNFs. The CV curves almost overlap during the subsequent cycles, indicating that it has good stability. Figure 6b shows the charge and discharge curves at the 1st, 50th and 100th cycles, it can be seen that an obvious platform appears at 0.6 V during the discharge processes, which corresponds to the conversion reaction at 0.55 V in the CV curve. The galvanostatic charge–discharge (GCD) process of CNF//MoS_2_-0.3 at the current density of 0.5 A g^−1^ is depicted in Figure 5d and an initial discharge capacity of 1342 mA h g^−1^ with the initial coulombic efficiency of 60% can be calculated based on the curve. The serious irreversible capacity loss in the first cycle may be caused by the side reactions, e.g., electrolyte decomposition and SEI formation. In the following cycles, the coulombic efficiency reaches to about 100% and no obvious attenuation has been observed, which reveals from the other side that the composite fibers possess a stable structure. The two-dimensional and layered structure of MoS_2_ nanosheets has a high specific surface area and contains a large number of active sites on the surface and edges, which enables Li^+^ to be stored in a two-step process and ensures a higher theoretical capacity [44,45,46]. By the comparison of GCDs with pristine CNFs (see Figure S4), the high capacity of the composite fibers can be ascribed to the synergistic effect between CNFs and MoS_2_ in CNF//MoS_2_ which enhances the further the electrochemical performance of the composites. The uniform anchoring of MoS_2_ nanosheets on the CNFs substrate could stabilize the structure of MoS_2_ with highly exposed active sites and a large specific surface area. The porous characteristic of the composite fiber and the high conductivity of the CNFs substrate can significantly improve the ionic and electronic conductivities. With the stepping increase in the applied current density from 0.2 to 5 A g^−1^, the CNF//MoS_2_-0.3 sample delivers a higher capacity than the other four samples (see Figure 6c and Figure S6). Even though the capacity decays with the increase in the applied current density, it could restore the original one when the current density returns to 0.2 A g^−1^, displaying a superior rate ability. After 200 charge–discharge cycles at 0.5 A g^−1^, CNF//MoS_2_-0.3 can maintain a capacity of 866.5 m Ah g^−1^ (see Figure 6d), showing an excellent Li^+^ storage capacity and good cycling stability. Meanwhile, the capacity for CNF//MoS_2_-0.05, CNF//MoS_2_-0.1, CNF//MoS_2_-0.15, CNF//MoS_2_-0.2 and CNF//MoS_2_-0.4 is 467.3, 549.0, 631.0, 768.2 and 696.7 mA h g^−1^, respectively, after 200 cycles at the same current density (Figure S7). Under a current density of 1 A g^−1^, a high specific capacity of 703.0 mAh g^−1^ can be maintained for CNF//MoS_2_-0.3 after 500 cycles (see Figure 6e). Both the specific capacity and the capacity retention of CNF//MoS_2_-0.3 are more advantageous than the other samples (Figure S8), indicating that the increase in the thiourea content can enhance the cycle stability of the composite fibers as an anode for LIBs. In addition, electrochemical impedance spectroscopy (EIS) confirmed the excellent conductivity of CNF//MoS_2_-0.3 (Figure 7a), which is in good agreement with the results of the rate performance (Figure 6c). EIS tests were performed for the prepared samples before cycling and for the CNF//MoS_2_-0.3 sample after 8 and 160 charge–discharge cycles. The Nyquist curve is composed of a semicircle in the high-frequency area and a straight line in the low-frequency part, where the diameter of the semicircle represents the electron transfer resistance and contact resistance and the slope of the straight line stands for the diffusion resistance of Li^+^ [47]. It can be seen from Figure 7a that the semicircles with diameters from a few tens of ohms for CNF//MoS_2_-0.05 to more than twenty thousand ohms for CNF//MoS_2_-0.15 have been depicted, indicating the distinction of the internal resistance for the six samples before cycling as an anode for LIBs. The low resistance of CNF//MoS_2_-0.05 is reasonable since it consists mainly of highly conductive carbon fibers. The high internal resistance of CNF//MoS_2_-0.15 might be due to the irregular or loose coating of the MoS_2_ nanosheet on the surface of CNFs, which induces more contact resistance in the composite fibers. EIS tests on the batteries using CNF//MoS_2_-0.3 as the anode at different cycles have been conducted. As can be seen from Figure 7a,b, the internal resistance of CNF//MoS_2_-0.3 underwent a dramatic decline after eight cycles of charge and discharge, and the diameters of the semicircle shrank from roughly 11,000 to about 70 Ω. Such a phenomenon can be explained due to the activation of the MoS_2_ nanosheets by the insertion of Li^+^ or the formation of SEI films, which strengthens the contact among the sheets and that between the MoS_2_ sheets and the CNFs substrates. The internal resistance increased slightly even after undergoing 200 cycles (see Figure 7b), indicating the stability of the structure and the reversibility of the electrochemical performance. The diffusion coefficient D of lithium ions can be estimated from Equations (3) and (4) [48].

Z′ = R_s_ + R_ct_ + σω^−1/2^ (3)

D = fR^2^T^2^/2A^2^n^4^F^4^C^2^σ^2^_w_ (4)

R is the gas constant, where the value is 8.314 J/(mol·K)^−1^, T is the thermodynamic temperature, where the value is 301.15 K, A is the electrode surface 5.0 × 10^−7^ m^2^, where the value is 2 × 10^−4^ m^2^, and n is the number of electrons transferred by a single molecule during the reaction, where the value is 4. F is the Faraday constant of 96,485 C mol^−1^, C is the Li^+^ concentration value of 1.2 × 10^4^ mol m^−3^ and σ_w_ is the Warburg coefficient. The slope of Z′ relative to the ω^−1/2^ line in the low-frequency range can be calculated by Equations (1)–(3) (ω represents the angular frequency, ω= 2πf, f is the frequency). As shown in Figure 7c, a slope of 21.61 can be deduced. Therefore, the diffusion coefficient of Li^+^ of about 1.28 × 10^−14^ cm^2^ S^−1^ has been estimated.

**Figure 8:**
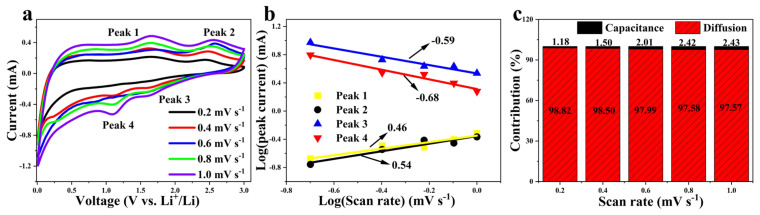
(**a**) CV curves under scan rates from 0.2 to 1.0 mV s^−1^; (**b**) curves of log(i) vs. log(ν) (b-value determination); (**c**) contribution ratios of the capacitive-related process at various scan rates for CNF//MoS_2_-0.3 sample.

In order to further explore the kinetic behavior of CNF//MoS_2_-0.3 as an anode for LIBs, the CV curves at different scan rates in the range 0.2~1.0 mV s^−1^ have been recorded. As shown in Figure 8, the pseudocapacitance contribution can be calculated according to the relationship between current (i) and scan rate (ν) [49,50].

i = aν^b^ (5)

log(i) = blog(ν) + log(a)(6)

where a and b are constants and the slope represents the b value, which can be calculated according to Equation (5) and its variation of Equation (6). It means diffusion is the dominant process for the electrochemical reaction when a b value close to 0.5 is obtained. Meanwhile, a pseudocapacitance-controlled process can be considered if the deduced value of b approaches 1. Figure 8b shows the dot plot of log (i) vs. log (ν) in the redox state. The b values calculated according to the two different oxidation peaks are of 0.46 and 0.54, respectively. Meanwhile, the values derived from the isolated reduction peaks are −0.59 and −0.68, respectively. Both the results indicate that the charge–discharge is mainly a diffusion-controlled reaction, which also corresponds to the obvious characteristics of the rate performance diagram. The capacitance contribution of CNF//MoS_2_-0.3 at a scan rate of 1.0 mV s^−1^ can be calculated through Equation (7) and its variation of Equation (8) [51].

i = k_1_ν + k_2_ν^1/2^ (7)

i/ν^1/2^ = k_1_ν^1/2^ + k_2_ (8)

The capacitance contribution of CNF//MoS_2_-0.3 at different scan rates is shown in Figure 8c. It can be seen that with the increases in the scan rate, the contribution of capacitance increases slightly from about 1.2% at a scan rate of 0.2 mV s^−1^ to only 2.4% at 1.0 mV s^−1^. Usually, the pseudocapacitive reaction on the surface is beneficial to the rate performance and cycle stability.

## 4. Conclusions

In summary, composite fibers consisting of MoS_2_ nanosheets anchored on carbon nanofibers (CNF//MoS_2_) have been successfully constructed by combining electrospinning with solvothermal reactions. Films of CNFs with diameters in the range of 200–300 nm are fabricated using the electrospinning method following a calcination step. They are utilized as a substrate and decorated by MoS_2_ nanosheets through a simple solvothermal reaction. The density of the MoS_2_ nanosheets anchored on the surface of CNFs can be tuned by changing the content of thiourea, which influences the growth of MoS_2_ by controlling the supply of S^2−^. The CNF skeletons of the films prevent MoS_2_ nanosheets from stacking into bulk ones and keep the porous structure of the composite fibers with a high specific surface area. Such a composite structure could shorten the diffusion distance of Li^+^ and promote the transfer of electrons when they are used as anodes for LIBs. It could also prevent the aggregation of MoS_2_ nanosheets during the charge–discharge cycles. The CNFs supply the composite fibers with high conductivity while the MoS_2_ sheets provide abundant active sites for reactions with Li^+^. The composite films can be used as self-supporting anodes for LIBs with the absence of binder and carbon black, which are required usually for the fabrication of electrodes. The synergistic effects between MoS_2_ and CNFs, along with the porous characteristics of the composite fibers, enhance the transfer of electrons and the diffusion of lithium ions. Therefore, a high reversible capacity of 866.5 mA h g^−1^ for 200 cycles at a current density of 0.5 A g^−1^ has been recorded with the composite fibers. It displays also excellent cycling stability for 500 cycles at 1 A g ^−1^ and maintains a capacity of 703.3 mA h g^−1^ after that. The combination of the facile solvothermal method and the easily operated electrospinning technology makes the large-scale application of CNFs//MoS_2_ composite fibers as self-supporting anodes for LIBs practicable.

## Data Availability

Data sharing is not applicable to this article.

## Supplementary Materials

The following supporting information can be downloaded at: https://www.mdpi.com/article/10.3390/nano13192689/s1, Figure S1: HRTEM images of the prepared samples; Figure S2: TGA curves of the composites; Figure S3: The N_2_ sorption isotherms and PSD curve of CNFs; Figure S4: CV, GDC and cycling performance for CNFs; Figure S5: CV profiles of CNF//MoS_2_-0.1, -0.15, -0.2 and -0.4; Figure S6: Rate performances of CNF//MoS_2_-0.05, -0.15 and -0.2; Figure S7: Cycling performances of CNF//MoS_2_-0.05, -0.1, -0.15, -0.2 and -0.4 at current density of 0.5 A g^−1^; Figure S8: Cycling performances of CNF//MoS_2_-0.05, -0.15 and -0.2 under current density of 1 A g^−1^.
