# Supplementary material for: Carbon Nanofibers Decorated by MoS2 Nanosheets with Tunable Quantity as Self-Supporting Anode for High-Performance Lithium Ion Batteries
Source: Nanomaterials (Basel). 2023 Sep 30;13(19):2689. doi: 10.3390/nano13192689 (PMC10574418; doi:10.3390/nano13192689)
Supplement: Supplementary file 1 [file nanomaterials-13-02689-s001.zip › nanomaterials-2606601-supplementary.pdf]

Supplementary Materials

Carbon Nanofibers Decorated by MoS_2 Nanosheets with Tunable Dispersity as Flexible Self-Supporting Anode for High-Performance Lithium Ion Batteries

Liyan Dang [1,†], Yapeng Yuan [1,†], Zongyu Wang [1], Haowei Li [1], Rui Yang [1], Aiping Fu [2], Xuehua Liu [1] and Hongliang Li [1,*]

[1] Institute of Materials for Energy and Environment, College of Materials Science and Engineering, State Key Laboratory of Bio-Fibers and Eco-Textiles, Qingdao University, Qingdao 266071, China
[2] College of Chemistry and Chemical Engineering, Qingdao University, Qingdao 266071, China
* Correspondence: lhl@qdu.edu.cn
† These authors contributed equally to this work.

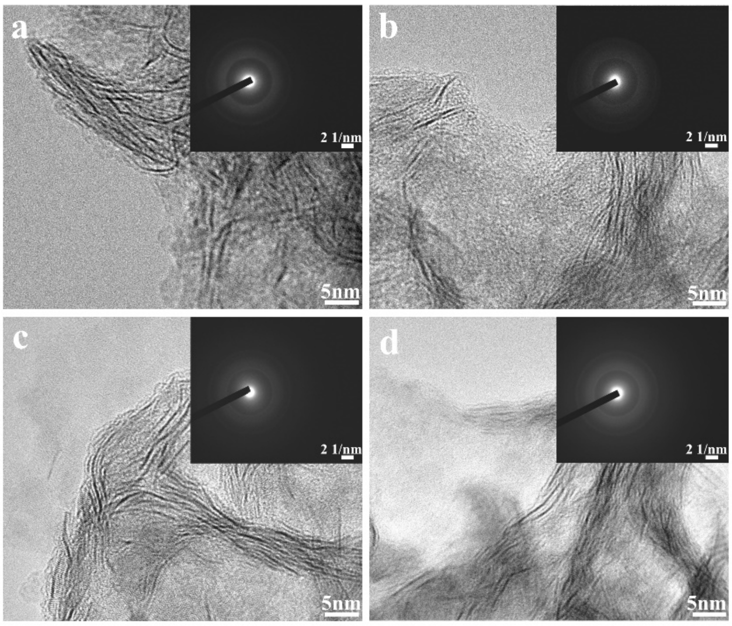

Figure S1. The HRTEM images of (a) CNF//MoS_2-0.1; (b) CNF//MoS_2-0.15;(c) CNF//MoS_2-0.2; (d) CNF//MoS_2-0.4, the insets show their SAED patterns.

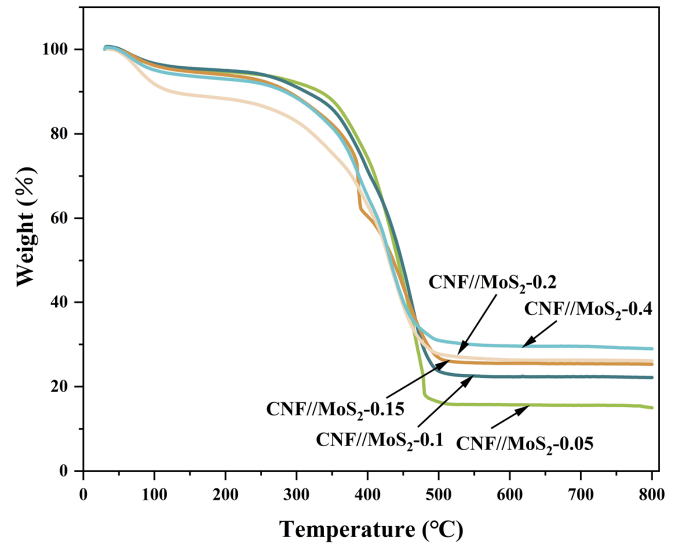

Figure S2. TGA curves of CNF//MoS_2-0.05, CNF//MoS_2-0.1, CNF//MoS_2-0.15, CNF//MoS_2-0.2, and CNF//MoS_2-0.4.

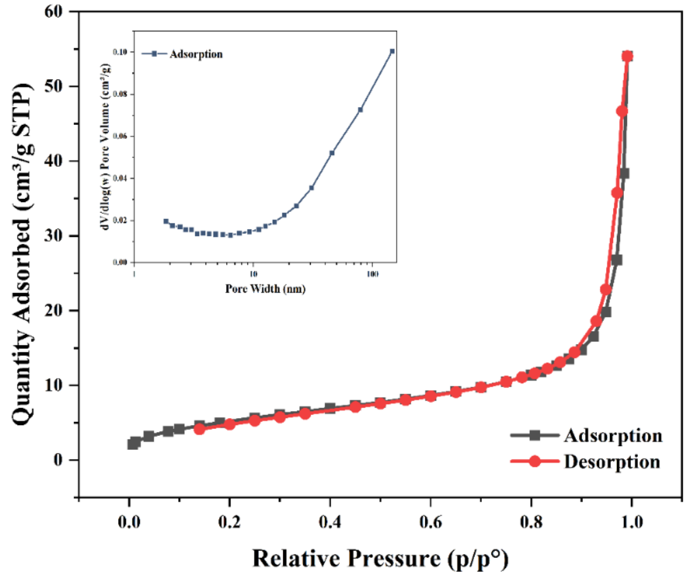

Figure S3. The N_2 sorption isotherms, and pore-size distribution curve (the inset) of CNFs.

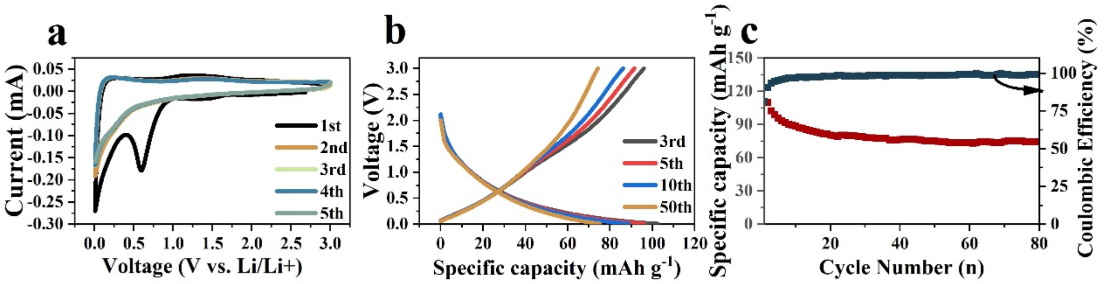

Figure S4. (a) CV profiles with a scanning rate of 0.2 mV s^{-1} and (b) GCD profiles at 0.2 A g^{-1} for

CNFs. (c) Cycling performance of CNFs at 0.2 A g^{-1}

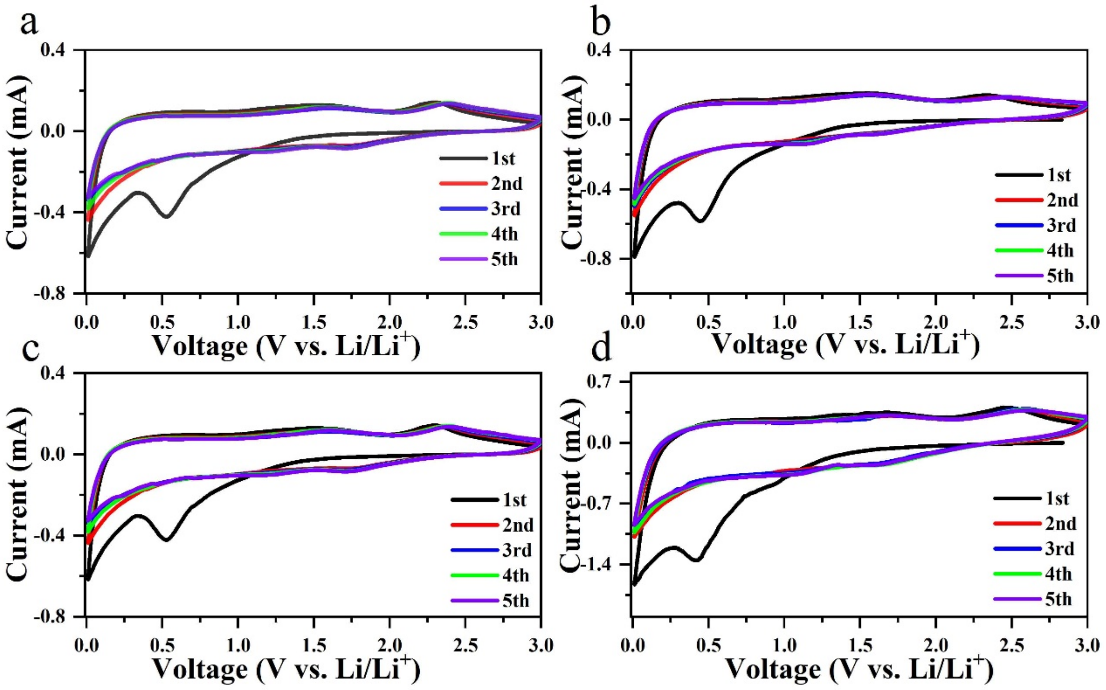

Figure S5. CV profiles of (a) CNF//MoS_2-0.1, (b) CNF//MoS_2-0.15, (c) CNF//MoS_2-0.2, (d) CNF//MoS_2-0.4, respectively.

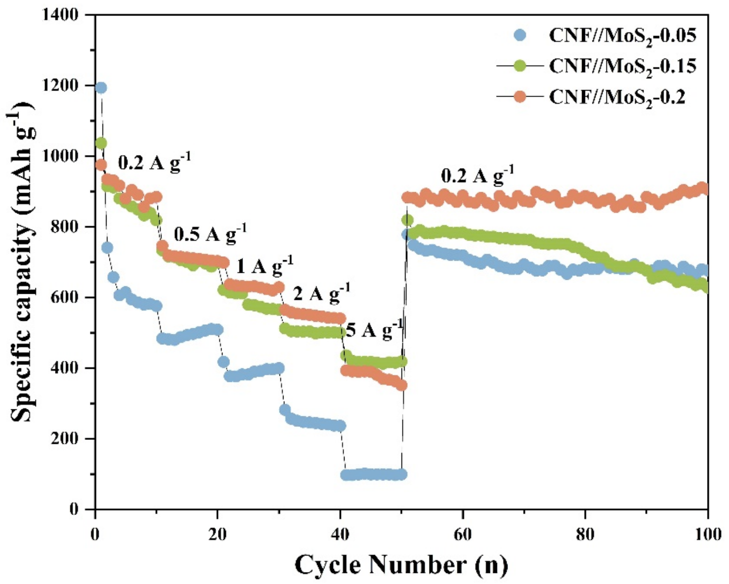

Figure S6. Rate performances of CNF//MoS_2-0.05, CNF//MoS_2-0.15 and CNF//MoS_2-0.2.

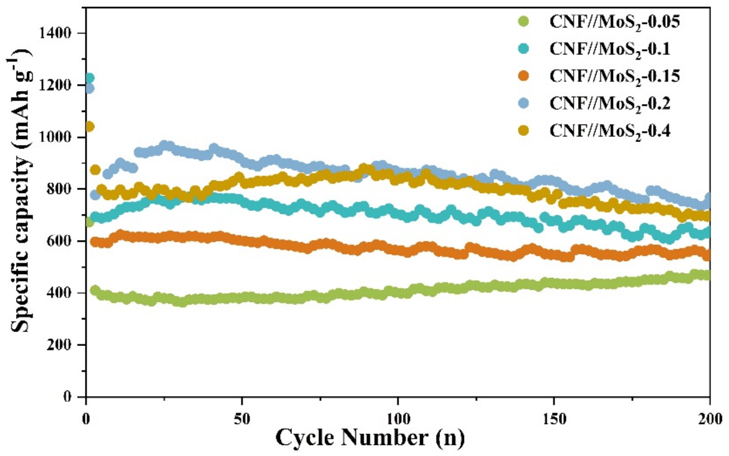

Figure S7. Cycling performances of CNF//MoS_2-0.05, CNF//MoS_2-0.1, CNF//MoS_2-0.15, CNF//MoS_2-0.2, and CNF//MoS_2-0.4 at current density of 0.5 A g^{-1}.

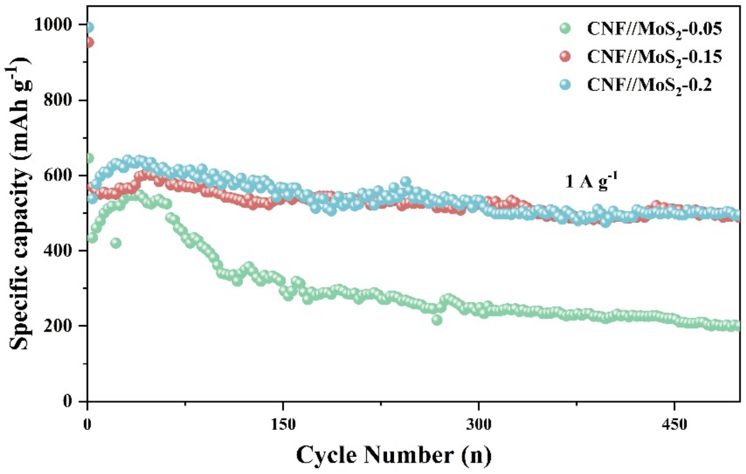

Figure S8. Cycling performances of CNF//MoS_2-0.05, CNF//MoS_2-0.15 and CNF//MoS_2-0.2 at current density of 1 A g^{-1}.
